# Inhibin-B and FSH Are Good Indicators of Spermatogenesis but Not the Best Indicators of Fertility

**DOI:** 10.3390/life12040511

**Published:** 2022-03-30

**Authors:** Katarzyna Jankowska, Natalia Suszczewicz, Michał Rabijewski, Piotr Dudek, Wojciech Zgliczyński, Radosław B. Maksym

**Affiliations:** 1 Department of Endocrinology, Centre of Postgraduate Medical Education, ul. Cegłowska 80, 01-809 Warsaw, Poland; kjankowska@cmkp.edu.pl (K.J.); pldudek@wp.pl (P.D.); wzgliczynski@cmkp.edu.pl (W.Z.); 2 Department of Mini-invasive and Endoscopic Gynecology, Military Institute of Medicine, ul. Zegrzyńska 8, 05-119 Legionowo, Poland; 3 Department of Reproductive Health, Centre of Postgraduate Medical Education, ul. Żelazna 90, 01-004 Warsaw, Poland; mirab@cmkp.edu.pl

**Keywords:** male infertility, inhibin B, sperm count, FSH, spermatogenesis, reproduction

## Abstract

Biochemical markers of spermatogenesis and fertility assessment are important in the practical management of infertile males and the determination of an individual’s prognosis. We performed an analysis on 100 males with a male infertility factor. The following study inclusion parameters were analyzed: seminogram, FSH, LH, testosterone, estradiol, prolactin, TSH, and inhibin B concentrations. The patients were subsequently treated by reproductive endocrinologists in accordance with AUA/ASRM and EAU guidelines. The reproductive status was evaluated over a period of 3 years. We found a strong correlation of sperm count with inhibin B (r = 0.74, *p* < 0.001) and FSH concentration levels (r = −0.46, *p* < 0.001). Among 95 patients at follow-up, pregnancies occurred for 59 of their partners (48 spontaneous, 5 after IVF–ET, and 6 after IUI). Thirty-six patients remained childless despite the therapy. Sperm count and inhibin B level were the best predictors of natural fertilization (ROC AUC: 0.86 and 0.84; cut-off: 2.7 mln/mL and 45 pg/mL). Although inhibin B and FSH can be used to evaluate spermatogenesis and fertility, the initial sperm concentration appeared to be the best predictor of success. Pregnancy was achieved in a surprisingly large proportion of patients with a very low concentration of inhibin B and a low initial sperm count. It is noteworthy that 81% of the pregnancies were achieved without medically assisted reproduction.

## 1. Introduction

The primary diagnostic tool used to assess male fertility is semen analysis. The most common indicator of male infertility is oligozoospermia, which is the decreased sperm count in semen. The sperm number should be ≥16 million/mL or ≥39 million sperm in the ejaculate [1]. The so-called oligoasthenoteratozoospermia (OAT) syndrome in infertile men involves a simultaneous drop in sperm concentration, motility, and morphology. It should be emphasized that the reference values for semen analysis were developed on the basis of an examination of males who were considered fertile by establishing the lower fifth percentile. Additionally, single deviations in the test results should not be equated with a diagnosis of male infertility, and results with numerous deviations are of the greatest diagnostic importance [2]. Increasing the sperm count and improving the sperm quality enhance the chance of conceiving naturally and increase the percentage of successful medically assisted reproduction (MAR) procedures, including in vitro fertilization (IVF). An interesting medical problem is the relationship between fertility and the results of semen tests in men with spermatogenesis disorders. Moreover, it has not yet been established how the preliminary results of semen tests translate into the final effectiveness of a treatment and the determination of the optimal treatment method. The World Health Organization (WHO) guidelines are not intended to provide a fertility potential or, conversely, infertility status. Men whose semen outcomes fall below the lower reference levels could still be fertile, and values within the “normal” range do not guarantee fertility. The guidelines were not developed to predict the success rate of expectant management and MAR, although they are routinely used for this purpose. Semen analysis, which is fundamental in assessing male fertility, indicates whether the problem involves the male partner and can estimate the disorder’s severity. Semen analysis can also indicate that additional sperm tests are required, like bacterial culture or hormonal evaluation. A large overlap between fertile and infertile populations exists for the main sperm parameters. A seminogram can help to distinguish between fertile and infertile men but it does not have enough power to constitute a diagnostic tool for infertility. Fertility is multifactorial and relies on sperm features, coital frequency, sexual function and, to a high extent, concomitant female disorders [3].

Spermatogenesis is a complex, multi-stage process that depends on many factors. Hormonal factors, for example, follicle-stimulating hormone (FSH), luteinizing hormone (LH), testosterone, estradiol, prolactin, thyroid-stimulating hormone (TSH), and inhibin B, all play an important role. Recently, the special role of FSH and inhibin B has been emphasized. It was demonstrated that inhibin B is the relevant circulating form of inhibin in male fertility. Inhibin B is produced in males exclusively by Sertoli cells in the testis, provides negative feedback for FSH secretion, and seems to be an important marker of the functioning of the seminiferous tubules [4]. Inhibin B also exerts paracrine intratesticular effects [5]. Serum inhibin B shows a clear diurnal variation that is closely related to that of testosterone. The administration of FSH increases the secretion of inhibin B in healthy men. Treatment of infertile men with FSH, however, does not result in an unequivocal inhibin B increase. A clear inverse relationship between serum inhibin B and FSH levels was also observed. Serum inhibin B levels are strongly positively correlated with testicular volume and sperm counts [6]. Inhibin B and FSH together seem to be a more sensitive and specific marker of spermatogenesis than either one alone [7]. The assessment of testicular function typically involves an initial endocrine assessment in which serum FSH, total testosterone, LH, estradiol, prolactin, and TSH levels are routinely measured [2,8].

In some clinical situations, a tool that enables the evaluation of male fertility by means of blood tests, without performing a semen test, could be very helpful, for instance, in adolescents and men not yet trying to conceive a baby or in medical settings with no direct access to a semen analysis laboratory. It has been suggested that the measurement of inhibin B and FSH in serum could serve as a partial substitute of a semen test or fecundability in epidemiological assessments [9].

An independent clinical problem is how to select patients for whom the use of a hormonal stimulation of spermatogenesis will be beneficial and effective. Up to now, no standard cut-off points have been established for the seminogram to determine the fertility prognosis of a given male. There is no single blood or semen test that would reliably and unambiguously assess the activity of the seminiferous epithelium and the effectiveness of spermatogenesis stimulation. The main issues are the relation of hormonal levels to spermatogenesis and the establishment of prognostic and predictive factors that could facilitate proper counseling and management of patients.

The main purpose of the study was to examine the relationship between the plasma concentrations of hormones, especially inhibin B and FSH, and the semen functional parameters among men who were partners in an infertile couple and the prospective results of infertility treatment. The aim of the study was also to investigate the potential use of endocrine markers as prognostic factors. The research hypothesis assumed that, thanks to the examination of endocrine fertility indicators, it is possible to predict the chances of success at the very beginning of a diagnosis and to inform patients more reliably about their prognosis related to various methods of treatment. The analyses carried out in the presented study included: levels of FSH, LH, testosterone, estradiol, prolactin, TSH, inhibin B, testicular volume, and semen parameters at inclusion to treatment. A three-year follow-up after inclusion in the study was conducted regarding the achievement of pregnancies in couples. Based on the analysis, possible significant correlations and predictive values for a reduced concentration of the above-mentioned indicators and the success of infertility treatment were established.

## 2. Materials and Methods

### 2.1. Patients

The study was performed at the Department of Endocrinology and the Department of Reproductive Health, Centre of Postgraduate Medical Education (CPME), Warsaw, Poland, among men (age range 20–54 years, mean 35.1 ± 5.09 year) from November 2017 through September 2021. In total, 100 men with primary male infertility were recruited. The study was conducted according to the guidelines of the Declaration of Helsinki and according to national law, as well as to the Statute (72/2021) and Regulations of the local Review Board (Bioethics Committee) at the CPME, which do not require and do not provide separate approvals for non-sponsored, non-interventional, and observational studies. Informed consent was obtained from all participants. 

Infertility was defined as the inability of a man to make his partner conceive despite unprotected, regular intercourse for a period of >12 months, while primary infertility was defined as inability of a couple to conceive throughout time. The final diagnosis of male infertility was made after excluding female infertility. Patency of the fallopian tubes and ovulatory cycles was confirmed, and the female partners had no visible abnormalities at a gynecological physical examination and gynecological ultrasound analysis. Non-obstructive azoospermia (NOA) was defined as the absence of sperm due to non-obstructive causes in two consecutive semen analyses after centrifugation of the sample. A karyotype analysis was performed in patients with severe oligozoospermia (concentration below 5 mln/mL). The testis volume and the presence of varicocele were assessed using scrotal ultrasonography by an experienced ultrasound specialist.

The exclusion criteria were defined as follows: a visible genital malformation, hypothalamic–pituitary–gonadal axis disorders with a gonadotropin deficiency, overt hyperprolactinemia, thyroid function disturbances, cryptorchidism, impaired fertility due to obstructive, genetic, and/or infectious reasons or abnormalities, such as orchitis and varicocele, as well as recent or current use of medication that could impair the reproductive and/or hormonal system.

### 2.2. Semen Analysis

The collection of semen for diagnostics and the semen analysis were consistent with the guidelines of the WHO [1]. Semen samples were processed at the same andrology laboratory and were provided by masturbation after a suggested 2–5-day abstinence time, ejaculated into a sterile plastic container, and placed in an incubator (37 °C) during liquefaction. All the semen samples were analyzed with the use of a Sperm Class Analyzer (SCA; Casa System Microptic) for volume, sperm count, total sperm number, sperm concentration, total and progressive motility, vitality, and morphology. The sperm movement was graded as progressive motility (A + B), non-progressive motility (C), and immobility (D). The concentration of spermatozoa was confirmed manually (examination with phase-contrast optics in a Neubauer improved chamber at a magnification of 100× and 400×). The eosin-nigrosin staining method was used for the assessment of spermatozoa vitality. Sperm morphology was verified manually using an oil-immersion microscope lens with a 1000× objective to classify spermatozoa as normal or abnormal according to Krueger strict criteria. If no sperm were seen in the chamber, the samples were also centrifuged at 600× *g* for 10 min. The absence of sperm in the pellet was considered as a diagnosis of azoospermia. Semen quality abnormalities were defined as follows: oligozoospermia (concentration <15 million/mL), severe oligozoospermia (concentration <5 million/mL), cryptozoospermia (concentration <1 million/mL), asthenozoospermia (motility A + B < 32 percent), and teratozoospermia (morphology < 4 percent normal forms). 

### 2.3. Laboratory Measurements 

Fasting blood samples were collected between 8:00 and 10:00 a.m. form the antecubital vein. After centrifugation, the serum was collected and frozen at −70 °C until required for analysis purposes. The serum levels of total testosterone (TT), prolactin (PRL), LH, and FSH were measured with immunometric assays (Immulite 2000 and RIA CAC; Siemens Medical Solution, Malvern, PA, USA). The normal values were: TT, 12–28 nmol/L (sensitivity—4 ng/dL), LH, 2–6 mIU/L (sensitivity—0.05 mIU/mL), and FSH, 3–18 mIU/L (sensitivity—0.1 mIU/mL). The inhibin B levels were quantified in the serum using an enzyme-linked immunosorbent assay (ELISA). 

### 2.4. Treatment and Follow-Up

The patients included in the study were offered treatment consisting of issuing health-related recommendations, dietary recommendations, and pharmacological treatment, including hormonal stimulation of spermatogenesis in accordance with the recommendations of the American Society of Reproductive Medicine (ASRM), the European Association of Urology (EAU), and the decision of the treating physician (reproductive endocrinologist). After issuing the recommendations in an outpatient clinic, the patients had the opportunity to continue trying for spontaneous pregnancy or to be treated in an assisted reproductive center, where they were subjected to the MAR procedure in accordance with the medical recommendations and the patient’s personal decisions regarding the scope of treatment. Additionally, a follow-up was conducted. Information about the occurrence and outcome of pregnancy was obtained from the attending physicians and the patients themselves. The patients were followed up to 3 years from the inclusion to the study, regardless of the chosen method of treatment. 

### 2.5. Statistical Analysis

Before running the tests that assume a normal distribution of the data, we ran log transformations to reduce the skewness. The Kolmogorov–Smirnov, Lilliefors, and Shapiro–Wilk tests were used to assess the distribution of the data. Spearman’s correlations were determined between the obtained measurements. Receiver operating characteristic (ROC) curves were generated to determine the area under the curve (AUC) to evaluate the diagnostic ability of the ratios as prognostic markers. The Youden’s score and the Tangent method were used to detect the optimal sensitivity and specificity separately for each assessed parameter/measurement. All the statistical calculations were performed in the Statistica environment (data analysis software system, TIBCO Software, Palo Alto, CA, USA), version 13 with 5.0.85 extensions. Results with *p* < 0.001 and *p* < 0.05 were considered significant for correlation and ROC–AUC analysis, respectively.

## 3. Results

Based on the performed research and analysis of the obtained data, it was possible to show that the sperm count was significantly and positively correlated with inhibin B level (r = 0.75, *p* < 0.001). The lower the concentration of inhibin B, the lower the number of sperm present in the semen. There was also a relationship between the seminogram and the FSH levels—the higher the FSH levels, the lower the number of sperm per milliliter (r = −0.46, *p* < 0.001)). A positive correlation was also observed between the total testicle volume and the sperm count (r = 0.39, *p* < 0.001). There was a weak negative correlation between LH concentration and sperm count (r = −0.37, *p* < 0.001), as well as a weak positive trend towards a correlation between sperm count and testosterone levels (r = 0.31, *p* > 0.01) (Table 1).

There was no relationship between the number of sperm and the concentrations of estradiol, TSH, and prolactin. Additionally, inhibin B level was negatively correlated with FSH (r= −0.66, *p* < 0.001) and LH (r= −0.46, *p* < 0.001) concentrations. 

Furthermore, it was checked whether inhibin B concentration could be used to predict a low sperm count (i.e., lower than 15 mln/mL). The area under the ROC curve was 0.92, indicating good discriminatory properties. The optimal cutoff, computed using the Youden index, was 148, resulting in 88% specificity and 83% sensitivity. 

### Follow-Up Results

**Figure 1:**
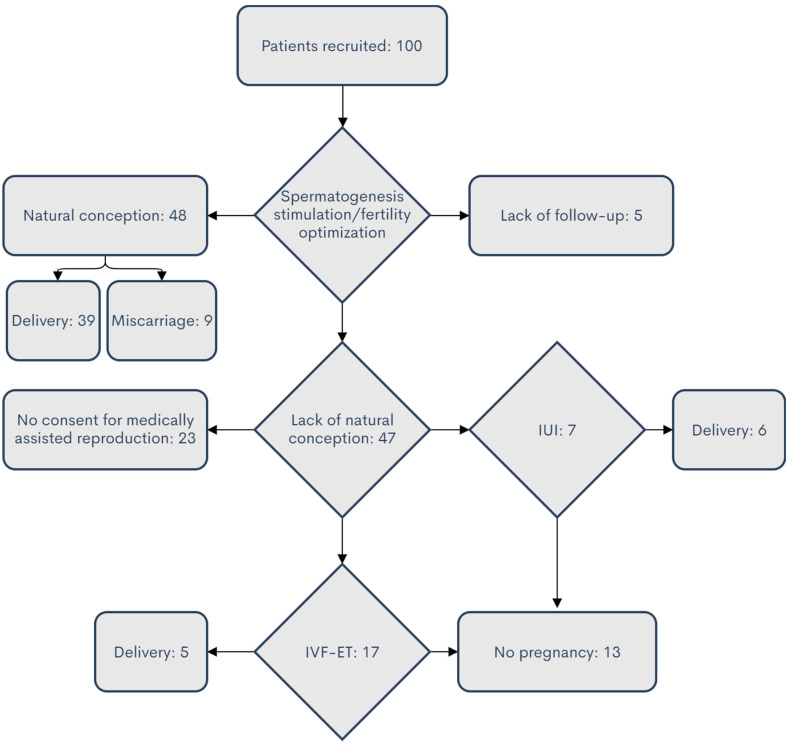
Reproductive status of the patients enrolled in the study at the end of the follow-up period. The number of patients in each category is indicated.

**Figure 2:**
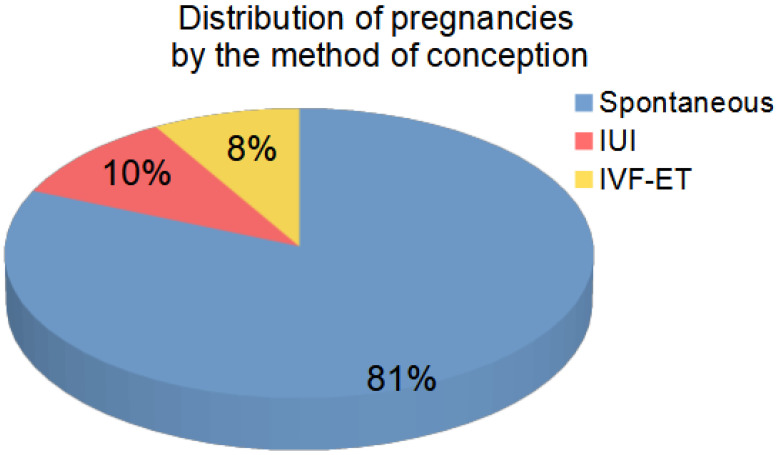
Percentage of pregnancies obtained in the course of the analysis depending on the method of conception.

The follow-up was not possible for five cases. Among 95 patients, pregnancies occurred in 59 cases (including spontaneously for 48 patients’ partners, after IVF for 5 of them, and after intrauterine insemination (IUI) for 6 of them). A total of 13 patients remained childless despite IVF or IUI procedures, and 23 couples decided that they did not want to use assisted reproductive methods (ART, i.e., IUI or IVF), despite the lack of spontaneous pregnancy. The distribution of the fate of patients participating in the analysis is shown in Figure 1 and Figure 2. Among the analyzed cases, 81% of the patients achieved pregnancy without the use of MAR methods. A summary of clinical data for patients is provided in Table A1. 

The obtained data and their accurate analysis indicated that pregnancies were sometimes achieved despite a very low concentration of inhibin B (the lowest concentration of inhibin B in cases of spontaneous pregnancy was 45 pg/mL, in cases of pregnancy after IUI, it was 78 pg/mL, and in cases of pregnancy after IVF, it was 34 pg/mL); on the other hand, pregnancies were not achieved despite normal FSH or inhibin B values. Thus, fertility was determined not only by hormone levels or even sperm parameters, but also by other factors related to both the man and his partner.

**Figure 3:**
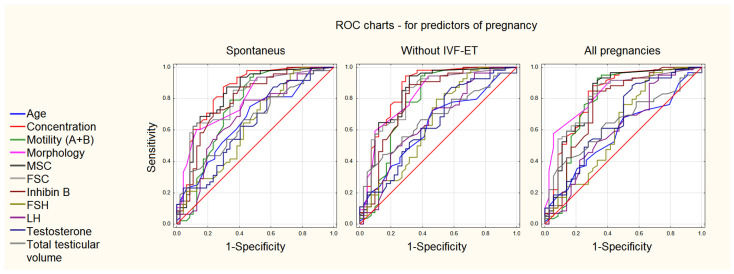
Comparison of the course of the ROC curves for selected and most significantly related clinical parameters in various groups of patients: spontaneous pregnancy (**left**), pregnancies without IVF–ET (**middle**), and all achieved pregnancies (**right**). The detailed parameters of the curves are presented in Table 2.

Although inhibin B and FSH levels can be used to evaluate spermatogenesis and fertility, the initial sperm concentration appeared to be the best predictor of success. The prediction analysis based on all the analyzed hormonal and sperm parameters using the ROC curve analysis is presented in Figure 3 and Table 2.

## 4. Discussion

In this study, we demonstrated a significant positive correlation between sperm count, serum levels of inhibin B, and testicular volume, on the one hand, and a negative correlation between sperm count and FSH levels, on the other. These results provide strong evidence that inhibin B, along with FSH and testicular volume, are important markers of the competence of Sertoli cells and spermatogenesis in a man, which is in accordance with the few reports to date on inhibin B and the quality of spermatogenesis [10,11,12,13]. In the cases of infertile men with elevated FSH levels, the correlation between inhibin B level and sperm count was more significant than that between FSH level and sperm count, as found in some publications [14]. This may be explained by the fact that inhibin is a direct product of the seminiferous tubules and that its secretion is stimulated in advanced stages of spermatogenesis [15]. In contrast, FSH levels are also affected by gonadotropin-releasing hormone (GnRH), estradiol, and testosterone. A further advantage of inhibin B measurement is that it reflects the function of the total testicular tissue, whereas a biopsy may not be representative of the entire testis and often shows a large variation in the completeness of spermatogenesis [16]. Although the data obtained in our study indicated that successful pregnancies are sometimes achieved despite a very low concentration of inhibin B, normal FSH or inhibin B levels are not a perquisite for success. They confirm that fertility is determined not only by hormone levels and sperm parameters but also by many other factors affecting a couple. New, unused and more accurate markers may be unveiled in the future, also including anti-Müllerian hormone (AMH) and insulin-like peptide 3 (INSL3) [17].

The treatment of male infertility is often long-lasting and complex, thus requiring andrological skills and experience. Treatment decisions may be obvious in some clinical situations. In clinical practice, however, there are circumstances where making a therapy decision is difficult. However, recent discoveries in the field of clinical genetics regarding gene polymorphism of FSH have shown that mutations of the FSH receptor and the androgen receptor have slightly changed the assessment of the results of hormone tests. For example, it turned out that patients with high FSH receptor sensitivity may have relatively low FSH concentrations despite oligozoospermia. In patients with high FSH receptor sensitivity, high levels of FSH are not required to activate the receptor. It seems that future advances in clinical genetics will make it possible to better plan the treatment of male infertility in the future. The assessment of genes related to the male reproductive function will, in some cases, be an invaluable indication of the treatment that should be applied to a given patient and of whether there are any chances of obtaining improvement [18,19]. 

All the described pregnancies were obtained from the partner’s sperm even when the initial parameters were poor. Thus, the results of the hormonal tests in these patients were not good indicators of fertility, even though the seminogram also showed numerous abnormalities, and the fertility prognosis was not good at the beginning of the therapy. In the next clinical trial, we plan to conduct additional genetic tests in such patients in order to help explain the effectiveness or ineffectiveness of the undertaken stimulation of hormonal spermatogenesis.

The obtained results and conclusions currently face certain limitations. The primary limitation of this study is that it was an observational, non-randomized study. Despite obtaining new conclusions, it would be worth repeating the work with more couples and a different population. Patient therapy was not homogeneous but adjusted depending on individual conditions, clinical experience, and the recommendations of reproductive endocrinologists. In addition, the decision regarding the treatment with MAR and ART methods was not made randomly or on the basis of medical indications but was an independent decision of the patients, made on the basis of various premises that we did not go into. It cannot be ruled out that patients who were reluctant to opt for the MAR and ART methods were more likely to undergo thorough diagnostics and long-term therapy. For ethical reasons, it would be a problem to design a control study with an untreated cohort. Confirmation of the results obtained in a presented observational study by follow-up and randomized controlled trials with a higher quality of evidence are warranted.

## 5. Conclusions

Hormones play a vital role in initiating and maintaining the male reproductive function. It seems that we can use the value of inhibin B to assess spermatogenesis. In oligozoospermia patients, inhibin B and FSH levels were highly correlated with sperm concentration. A decreased concentration of inhibin B correlated with the number of sperm and the FSH level. High levels of FSH and reduced levels of inhibin B clearly indicated an impairment of the spermatogenic function. Neither FSH alone nor luteinizing hormone (LH) alone seemed to be reliable markers of testicular function. FSH and inhibin-B can be useful to assess spermatogenesis when the patient does not want to or is unable to perform a sperm analysis. An optimal level of inhibin B to assess male infertility has not yet been established. Partners’ pregnancies occurred even at very low concentrations of inhibin B and very high concentrations of FSH. It should, therefore, be considered that inhibin B and FSH are good indicators of spermatogenesis but not the best indicators of fertility. It should also be noted that, among the analyzed cases, 81% of patients achieved pregnancy without the use of MAR and ART methods. In addition, the use of MAR and ART methods after the failure of the treatment optimizing natural fertility was not associated with obtaining a significant number of additional pregnancies and deliveries. 

In conclusion, we confirmed a strong correlation of inhibin B and FSH levels with spermatogenesis. Our results provide that the measurement of serum inhibin B and FSH levels is still of limited clinical relevance for individual patients, and further studies are needed. We conclude that inhibin B is the best available endocrine marker of spermatogenesis in subfertile men. Inhibin B estimation may prove to be an alternative to testicular biopsy in the differentiation between normal and impaired spermatogenesis. The obtained results, after further verification, may be used to change therapeutic strategies and provide more reliable counseling to patients entering treatment.

## Figures and Tables

**Table 1:** Correlation coefficients of clinical measurements with semen parameters.

Spearman’s Rank Correlation			
	**Concentration**	**Motility A + B**	**Morphology**
inhibin B	0.742	0.535	0.486
FSH	−0.464	−0.396	−0.309
LH	−0.370	−0.330	−0.248
testosterone	0.309	0.366	0.377
Total testicular volume	0.397	0.369	0.371

Significant correlations (*p* < 0.01) are shown in red.

**Table 2:** Results of AUC–ROC analysis of predictors of successful pregnancy.

AUC ROC Analysis of Predictors																		
	Spontaneus Pregnancies						Pregnancies without IVF-ET						All Pregnancies					
	AUC	SE	CI (95%)		Optimal cut-off		AUC	SE	CI (95%)		Optimal cut-off		AUC	SE	CI (95%)		Optimal cut-off	
predictor					Youden	Tangent					Youden	Tangent					Youden	Tangent
Concentration	0.844	0.041	0.763	0.926	2.7	2.7	0.861	0.042	0.779	0.942	1.8	1.8	0.820	0.048	0.725	0.914	1.8	1.8
MSC	0.827	0.044	0.740	0.913	45.0	45.0	0.841	0.044	0.754	0.928	9.6	9.6	0.815	0.050	0.717	0.913	9.6	9.6
FSC	0.797	0.047	0.706	0.889	342.0	240.0	0.820	0.046	0.730	0.910	45.0	32.2	0.802	0.049	0.706	0.899	10.2	10.2
Normal Morphology	0.789	0.046	0.699	0.880	3.0	3.0	0.820	0.045	0.732	0.908	1.0	1.0	0.834	0.042	0.751	0.917	2.8	1.0
Inhibin B	0.786	0.048	0.693	0.879	120.0	89.0	0.795	0.050	0.697	0.893	78.0	78.0	0.758	0.056	0.648	0.868	78.0	78.0
Motility A + B	0.743	0.053	0.638	0.847	5.0	3.4	0.773	0.054	0.666	0.879	5.0	5.0	0.786	0.057	0.674	0.897	5.0	5.0
LH	0.680	0.056	0.571	0.790	4.2	4.2	0.653	0.058	0.540	0.767	4.2	6.3	0.612	0.062	0.491	0.733	5.6	6.3
Age	0.663	0.056	0.553	0.773	33.0	33.0	0.625	0.059	0.510	0.740	33.0	33.0	0.581	0.061	0.462	0.700	33.0	28.0
Testicular volume	0.657	0.056	0.548	0.766	36.0	22.0	0.673	0.055	0.565	0.780	31.0	22.0	0.650	0.056	0.540	0.760	31.0	18.6
Testosterone	0.635	0.057	0.523	0.746	3.2	3.2	0.645	0.058	0.532	0.759	3.2	3.2	0.612	0.062	0.491	0.733	3.2	3.2
FSH	0.631	0.057	0.519	0.744	10.7	10.7	0.647	0.059	0.531	0.764	10.7	14.4	0.620	0.063	0.495	0.744	13.8	13.8
TSH	0.569	0.061	0.450	0.688	1.3	1.3	0.558	0.060	0.441	0.675	1.3	2.0	0.510	0.060	0.392	0.629	1.3	4.7
Prolactin	0.521	0.062	0.400	0.642	13.7	13.7	0.578	0.061	0.458	0.698	15.9	13.7	0.602	0.061	0.482	0.722	13.7	5.7
Estradiol	0.520	0.060	0.402	0.638	35.7	34.7	0.570	0.060	0.453	0.687	35.7	10.4	0.612	0.060	0.495	0.729	34.6	10.4

Significant predictors (*p* < 0.05) are shown in red. SE—standard error. CI (95%)—95% confidence interval.

## Data Availability

Data supporting the reported results can be obtained from the corresponding authors.

## Appendix A

**Table A1:** Summary of the outcomes of hormonal measurements, seminogram parameters, and follow-up results.

No.	FSH IU/L	LH IU/L	Testosterone ng/mL	INHIBIN B pg/mL	The Volume of Both Testicles	Sperm Concentration (mln/mL)	Follow-Up
1	22	10	3.8	36	15	0.1	F
2	3.4	2.3	4.4	179	23	8	A
3	13.8	3.5	3.9	45	15	0.1	A
4	0.4	0.5	0.27	13	16	0	C
5	22	16.8	2	9	18	0.1	D
6	13.4	2.4	2.2	121	15	11	A
7	11.8	6.5	4.6	73	27	4.6	C
8	3.2	2.8	7.4	139	17	3	A
9	10.5	3.9	3.2	159	12	18.7	A
10	5.8	1.8	3.2	121	16	0.4	C
11	29.5	3.3	3.1	20.4	6	0.1	D
12	6.1	1.6	3.2	49	17	6.2	C
13	4.8	1.2	1.6	139.2	18.6	31	A
14	34.2	9.9	2.1	5	9.6	0	D
15	4.2	2.5	4	97	22.4	2.3	A
16	9.8	3.9	3.4	124	19	4.6	A
17	38	13	2.28	3	6.2	0	C
18	13.3	4.4	1.57	9	10	0	C
19	12.4	3.7	2.43	90	25	11	A
20	8.6	5.4	5.3	170	27	14	A
21	23.1	7	5.45	35	19.8	0.1	D
22	15.8	3.9	2.9	48.5	13	0.1	C
23	9.6	2.3	4.6	154.92	26.9	58	B
24	1.5	2	3.2	263.57	18.6	148	A
25	7.6	2.5	0.6	9.2	16.7	0.1	C
26	4.4	3.5	2.64	99	26.1	0	C
27	3.7	1.6	3	235	30.3	40	D
28	5.9	3.3	4.05	125	21.5	9	D
29	2.2	2	3.34	183	26.8	5	A
30	3.2	3.2	5.2	164.3	42.2	20	A
31	4.3	2.5	4.98	493	15.08	94	A
32	13.2	3.3	5	92.8	32	14.1	B
33	1.3	1.1	2.9	211.4	17.1	17	A
34	11.4	2.8	5.6	75	17.7	0.1	C
35	23.9	8.6	2.67	29	17	0	D
36	4.07	1.55	2.87	158	34	29	G
37	15.9	5.6	2.4	56	17	14.2	A
38	2.2	2.15	4.4	276	19	258	A
39	6.8	3.5	3.2	89	22	1.8	A
40	10.2	5.7	4.1	58	18.5	1.6	C
41	10.9	3.5	6.6	69	27	2	D
42	7.6	1.91	4.1	123	22	4.5	D
43	8.3	2.5	7.5	142	34	2.7	B
44	11.3	4.2	11.6	120	16	5.1	A
45	14.8	7.9	2.8	46	20	0.9	C
46	19.8	7.5	7.6	58	34	6.4	C
47	7.7	2.9	5.2	51	27	0.8	B
48	37.2	7.1	4.5	7	19	0	C
49	3.9	6	6.7	209	30	10	C
50	6.8	4.3	2.9	63	21.1	0.1	D
51	2.6	0.94	3.9	161	47	6	B
52	7	7.3	4.7	149	37	21	A
53	14.4	5	3.1	108	42	3	B
54	8.42	4.9	2.1	205.6	20	0.1	C
55	4.3	2.76	5	157	33	14.4	A
56	2	3	2.84	171	33	0	C
57	14	7	4.2	139.4	21	0.1	D
58	17.1	4.4	7	58	15.1	0.1	C
59	1.83	2.04	4.3	612	41	94	A
60	11.6	4.5	3.18	82.7	8	0.6	F
61	3.5	2.1	5.22	207	37.7	20	B
62	9.3	7.2	5	161	37	50	A
63	10.8	3.2	2.77	53	17.99	0.02	C
64	2.86	2.36	4.1	260	38	66	C
65	8.56	2.69	4.86	145	37	10	A
66	9.6	4.3	5.08	112	30	5.2	G
67	8.7	2.2	5.7	123	27	7.8	A
68	6.4	2.5	8.82	156	31	14.7	A
69	9.7	6.2	4.3	127	0	9.7	A
70	12.9	3.2	4.92	59	34	0.7	F
71	10.7	3.7	7.3	98	45	4.9	B
72	7.9	1.8	9.6	145	41	12.3	H
73	8.4	2.1	8.32	176	21	14.1	A
74	6.9	2.6	8.28	124	32	9.8	A
75	11.3	5.2	5.8	98	14	2.3	G
76	10.7	4.8	4.05	127	22	5.8	A
77	12.8	5.4	3.73	45	19	0.9	B
78	7.9	3.1	7.28	67	43	1.9	A
79	8.6	2.9	5.46	78	39	4.9	A
80	6.2	4.2	4.99	178	0	11.8	H
81	7.8	1.8	10.2	220	51	15	A
82	12.8	6.3	3.51	147	21	10.8	B
83	5.9	3.6	7.47	124	47	15.3	C
84	8.5	3.2	5.27	78	50	11.4	A
85	11.8	5.4	3.35	54	24	6.4	A
86	9.7	4.9	8.03	62	0	8.6	H
87	9.1	4.8	4.58	34	32	5.2	C
88	7.8	2.1	9.32	179	45	11.7	A
89	11.9	9.4	4.33	53	21	7.9	H
90	6.6	3.5	7.09	197	36	12.6	D
91	9.5	5.2	5.8	78	41	8.5	G
92	8.9	5.3	5.8	34	23	0.2	F
93	12.5	2.1	5.18	48	26	0.7	D
94	6.7	6	3.79	95	41	2.1	G
95	7.4	2.7	4.61	123	53	9.5	A
96	4.5	4.4	6.65	154	46	12.2	H
97	7.3	5.2	3.73	112	0	2.3	G
98	7	2.9	5.24	52	30	0.9	E
99	6.4	5.2	5.93	98	20	3.4	F
100	8.2	4.5	4.8	44.2	25	0.7	D

Follow-up categories: A—spontaneous pregnancy and delivery, B—spontaneous pregnancy and miscarriage, C—no pregnancy, IVF/IUI was not applied, D—no pregnancy after IVF, E—no pregnancy after IUI, F—pregnancy after IVF, G—pregnancy after IUI, H—lack of follow-up.

## References

[1] World Health Organization (2021). WHO Laboratory Manual for the Examination and Processing of Human Semen.

[2] Schlegel P.N., Sigman M., Collura B., De Jonge C.J., Eisenberg M.L., Lamb D.J., Mulhal J.P., Niederberger C., Sandlow J.I., Sokol R.Z. (2021). Diagnosis and Treatment of Infertility in Men: AUA/ASRM Guideline Part I. J. Urol..

[3] Oehninger S., Ombelet W. (2019). Limits of current male fertility testing. Fertil. Steril..

[4] Pierik F.H., Vreeburg J.T., Stijnen T., De Jong F.H., Weber R.F. (1998). Serum inhibin B as a marker of spermatogenesis. J. Clin. Endocrinol. Metab..

[5] Barbotin A.L., Ballot C., Sigala J., Ramdane N., Duhamel A., Marcelli F., Rigot J.-M., Dewailly D., Pigny P., Mitchell V. (2015). The serum inhibin B concentration and reference ranges in normozoospermia. Eur. J. Endocrinol..

[6] Meachem S.J., Nieschlag E., Simoni M. (2001). Inhibin B in male reproduction: Pathophysiology and clinical relevance. Eur. J. Endocrinol..

[7] Kong X., Ye Z., Chen Y., Zhao H., Tu J., Meng T., Xiong C., Li H., Gong Y., Zheng L. (2021). Clinical application value of Inhibin B alone or in combination with other hormone indicators in subfertile men with different spermatogenesis status: A study of 324 Chinese men. J. Clin. Lab. Anal..

[8] Anawalt B.D. (2013). Approach to male infertility and induction of spermatogenesis. J. Clin. Endocrinol. Metab..

[9] Meeker D.J., Godfrey-Bailey L., Hauser R. (2007). Relationships between serum hormone levels and semen quality among men from an infertility clinic. J. Androl..

[10] Illingworth P.J., Groome N.P., Byrd W.I.L.L.I.A.M., Rainey W.E., McNeilly A.S., Mather J.P., Bremner W.J. (1996). Inhibin-B: A likely candidate for the physiologically important form of inhibin in men. J. Clin. Endocrinol. Metab..

[11] Anawalt B.D., Bebb R.A., Matsumoto A.M., Groome N.P., Illingworth P.J., McNeilly A.S., Bremner W.J. (1996). Serum inhibin B levels reflect Sertoli cell function in normal men and men with testicular dysfunction. J. Clin. Endocrinol. Metab..

[12] Jensen T.K., Andersson A.M., Hjollund N.H.I., Scheike T., Kolstad H., Giwercman A., Henriksen T.B., Ernst E., Bonde J.P., Olsen J. (1997). Inhibin B as a serum marker of spermatogenesis: Correlation to differences in sperm concentration and follicle-stimulating hormone levels. A study of 349 Danish men. J. Clin. Endocrinol. Metab..

[13] Klingmüller D., Haidl G. (1997). Inhibin B in men with normal and disturbed spermatogenesis. Hum. Reprod..

[14] Andersson A.M., Toppari J., Haavisto A.M., Petersen J.H., Simell T., Simell O., Skakkebæk N.E. (1998). Longitudinal reproductive hormone profiles in infants: Peak of inhibin B levels in infant boys exceeds levels in adult men. J. Clin. Endocrinol. Metab..

[15] Klaij I.A., Van Pelt A.M.M., Timmerman M.A., Blok L.J., De Rooij D.G., De Jong F.H. (1994). Expression of inhibin subunit mRNAs and inhibin levels in the testes of rats with stage-synchronized spermatogenesis. J. Endocrinol..

[16] Tournaye H., Verheyen G., Nagy P., Ubaldi F., Goossens A., Silber S., Van Steirteghem A.C., Devroey P. (1997). Are there any predictive factors for successful testicular sperm recovery in azoospermic patients?. Hum. Reprod..

[17] Babul-Hirji R., Hirji R., Chitayat D. (2021). Genetic counselling for infertile men of known and unknown etiology. Transl. Androl. Urol..

[18] Velez D., Hwang K. (2020). Personalized Medicine for the Infertile Male. Urol. Clin. N. Am..

[19] Sansone A., Kliesch S., Isidori A.M., Schlatt S.A.M.H. (2019). AMH and INSL3 in testicular and extragonadal pathophysiology: What do we know?. Andrology.

